# Research progress on human papillomavirus-negative cervical cancer: A review

**DOI:** 10.1097/MD.0000000000039957

**Published:** 2024-10-11

**Authors:** Ning Shao

**Affiliations:** a Department of Obstetrics and Gynecology, Xinhua Hospital, Shanghai Jiaotong University School of Medicine, Shanghai, China.

**Keywords:** cervical cancer, HPV-independent, molecular genetics

## Abstract

Cervical cancer is the fourth most common cancer in women worldwide. The vast majority of cervical cancers are associated with human papillomavirus (HPV) infection, but a small proportion of cervical cancers occur independently of HPV infection, with different subtypes having varying rates of occurrence. Despite the presence of false negatives in current testing, improving the accuracy of detection is crucial for studying the pathogenesis of HPV-negative cervical cancer and improving the prognosis of these patients. Existing research suggests that HPV-negative cervical cancer has a different pathogenesis from HPV-positive cervical cancer, although the exact mechanism is not yet clear. It is currently believed to be associated with the immune microenvironment, certain tumor gene mutations, and some long noncoding RNAs. This article provides an overview of the latest research progress on HPV-negative cervical cancer, including possible reasons, pathogenesis, pathological features, and clinical characteristics, aiming to provide new insights for diagnosis, treatment, and prognosis improvement.

## 1. Introduction

Cervical cancer is the fourth most common cancer in women worldwide, accounting for 6.5% of all cancers. The mortality rate of cervical cancer patients ranks fourth among all cancers, accounting for 7.5% of all female cancer-related deaths.^[1]^ Over 90% of cervical cancers are caused by human papillomavirus (HPV) infection.^[2]^ In the United States, due to inadequate screening, an estimated 14,000 people are diagnosed with cervical cancer each year, with over 4000 deaths attributed to cervical cancer. The majority of global cases are in low- and middle-income countries.^[3]^ Therefore, it is essential to not only increase HPV vaccination and effective screening but also develop treatment methods for women already diagnosed with the disease.^[3,4]^ The prevalence of HPV-positive cervical cancer significantly increased from 85.9% to 92.9% between 1990 and 2010.^[5]^ This increase is closely related to the improvement and widespread adoption of cervical cancer screening technologies and advancements in pathological tissue diagnosis. However, there are still 3% to 8% of cervical cancers that are truly HPV-negative.^[6–8]^

Compared to HPV-positive cervical cancer patients, HPV-negative cervical ADC is more common. Due to the difficulty of early screening, diagnosis is often made in the advanced stage with lymph node involvement, resulting in a poorer prognosis. The molecular etiology of HPV-negative ADC is not yet clear, but some studies suggest that mutations in TP53, PIK3CA, and CDKN2A may be associated with its pathogenesis.^[9–12]^ For HPV-negative cervical cancer, there is currently no specific treatment available. It is important to explore its pathogenesis, identify effective treatment options, and provide more treatment choices for these patients to improve their poor prognosis. In this review, we will discuss the latest research advances in HPV-negative cervical cancer, including possible causes, pathogenesis, pathological features, clinical characteristics, and more, to provide new ideas for diagnosis, treatment, and prognosis improvement.

## 2. HPV-negative cervical cancer possible causes

### 2.1. Tumor misclassification

Endometrial cancer development involving the cervix or other sites causing distant metastasis of HPV-negative tumors may lead to misclassification as HPV-negative cervical cancer. Hopenhayn et al found in their study that over 50% of cases could not be distinguished solely based on histological features from endometrial cancer.^[13]^ Therefore, for HPV-negative cervical specimens, further differentiation can be achieved by immunohistochemical staining of tumor and stroma. For example, negativity for estrogen receptor (ER), progesterone receptor (PR), P16, and CD10, as well as positivity for carcinoembryonic antigen (CEA), diffuse p16, CD34, and HPV, suggests adenocarcinoma. Conversely, positivity for ER, PR, P16, diffuse p16, and CD10, as well as negativity for CEA, CD34, and HPV, suggests endometrial adenocarcinoma.^[14,15]^

### 2.2. HPV infection latency period

There is a latency period from HPV infection to the appearance of cervical cell changes. During this period, the immune system suppresses the virus, preventing active replication. This can lead to a higher chance of false negatives due to low HPV DNA load, even in HPV-positive patients, which may not be detected by current HPV testing methods. A study spanning 5 years showed that, apart from cases with precancerous lesions or disease progression, most HPV infections disappear within 2 years.^[16]^

### 2.3. Errors in HPV detection methods

Current HPV detection methods can be classified into nucleic acid and nonnucleic acid signal amplifications. Nucleic acid signal amplification includes polymerase chain reaction and transcription-mediated amplification, such as Cobas HPV, APTIMA HPV. Nonnucleic acid signal amplification methods include hybrid capture and signal amplification, such as Hybrid Capture 2, Cervista HPV HR, Cervista HPV16/18. Compared to nucleic acid signal amplification methods, nonnucleic acid signal amplification methods have lower sensitivity and higher thresholds, leading to a higher potential for false-negative results, especially when the viral load in the sample is low.^[17]^ In HPV testing, the HPV L1 sequence is often used as a primary target sequence due to its high conservation across different HPV genotypes. Other sequences targeted for detection include E1, E2, E6, E7, and L2. However, during transcription to the host, these gene segments may be lost. If HPV detection targets these lost segments, false-negative results may occur.^[18,19]^ Current HPV tests primarily focus on screening for high-risk HPV types, limiting the detection of non-high-risk HPV types that may also contribute to cervical cancer. This limitation can result in some cases of false-negative results in HPV testing.

### 2.4. Sampling error

In addition to the detection method used, false-negative results in HPV testing can also be attributed to sampling errors. Small lesions may not yield cells from the relevant lesion site, sampling from inflamed or necrotic areas can result in poor cell viability, prolonged specimen transport times can lead to cell degradation, and the presence of blood or other chemical agents in the sample can also contribute to false negatives. Therefore, in cases of cervical cancer with negative HPV testing, alternative testing methods based on different principles should be considered for further validation.^[17,20]^

## 3. Mechanisms of HPV-negative cervical cancer

As 1 of the common malignant tumors in females, cervical cancer is primarily caused by HPV infection. Current research primarily focuses on HPV-positive cervical cancer. However, a small percentage of cervical cancers occur without detectable HPV infection. The exact mechanisms underlying the development of these HPV-negative cervical cancers are not yet fully understood. Current studies suggest that they may be associated with the immune microenvironment, tumor gene mutations, and certain long noncoding RNAs (lncRNAs).

### 3.1. Immune microenvironment

Evans and colleagues compared the immune microenvironment of HPVα9 segment (similar to HPV16), HPVα7 (similar to HPV18), and HPV-negative cervical cancer. They found that the level of lymphocyte infiltration is significantly lower in HPV-negative cervical cancer. In addition to lower levels of markers such as B, T, and NK lymphocytes, the expression of T cell effector molecules, activation/exhaustion markers, and T cell receptor diversity is also significantly reduced.^[21]^

In HPV-positive tumor tissues, CD4+ helper T cells, CD8+ cytotoxic T cells, and regulatory T cells (Tregs) exhibit a predominant Th2 humoral immune phenotype. The expression levels of these immune cells are increased, along with elevated levels of IFN-γ, TNF, perforin, and granzymes A, B, K. There is no significant difference in the expression of these molecules between HPVα9 and α7 tumor tissues. Studies have also found that HPV-positive cervical cancer expresses immune regulatory genes, including IDO and PDL1, at high levels, indicating a phenotype of tumor infiltration by T cells. In contrast, HPV-negative cervical cancer shows lower levels of immune cell infiltration and a lack of corresponding immune editing. However, studies have found that HPV-negative cervical cancer expresses more potential neoantigens. These results reflect significant differences in the immune environments of HPV-positive and HPV-negative cervical cancer, as well as appropriate differences between HPVα9 and α7 cervical cancer. These “differences” may lead to changes in patient outcomes between HPV-negative and HPV-positive cervical cancer, as well as potential changes in patient outcomes associated with cervical cancer related to different HPV types.^[21]^ The differences in immune microenvironments are helpful for distinguishing HPV-negative cervical cancer. The development of cancer is complex and may result from the combined effects of multiple pathways and factors. The clearer the understanding of the interaction between the tumor and its local immune microenvironment, the more personalized treatment plans can be developed for patients, leading to better prognoses.

### 3.2. Gene mutations in HPV-negative cervical cancer

Some studies have found that cervical cancer harbors many genes related to other tumors, including PTEN, TP53, KRAS, ERBB2, FAT1, and ARID1A. Among these, KRAS mutations are more common in adenocarcinomas, and high levels of activating mutations are associated with poor prognosis.^[22–25]^ In the development of HPV-related cervical cancer, somatic mutations occur in the cells, activating oncogenes and inactivating tumor suppressor genes. In this process, mutations in PIK3CA and the PI3K/Akt/mTOR signaling pathway play important roles.^[26,27]^ Some studies suggest that mutations in TP53, PIK3CA, and CDKN2A may be associated with the pathogenesis of HPV-negative cervical cancer.^[9–12]^

David Jenkins and colleagues conducted detailed pathological diagnosis on 45 ADC specimens. The results showed that 20 of them were typical ADC, with 17 being HPV-positive. The remaining 25 cases were of pathological types including endometrioid, mucinous, clear cell, and gastric types. Upon retesting the specimens for HPV DNA and performing immunohistochemistry, it was found that some had somatic mutations, including mutations in the STK11 system, which is particularly common in cervical gastric-type adenocarcinoma (GCA). These mutations were similar to those found in some HPV-positive typical ADC cases.^[28]^ A study on primary ADC identified a distinct subtype called cervical endometrioid-like adenocarcinoma, predominantly composed of HPV-negative tumors. The characteristic features of this subtype include a higher frequency of KRAS, ARID1A, and PTEN mutations.^[7]^ The discovery of these mutated genes helps to deepen the understanding of the pathological characteristics and biological behavior of HPV-negative cervical cancer, providing guidance in cervical cancer prevention. HPV-negative cervical cancer often has a poorer prognosis, and identifying specific gene mutations associated with cervical cancer can serve as potential diagnostic markers. This is beneficial for detecting and screening early-stage cancer, improving patient outcomes. Identifying key gene mutations also provides a reference for analyzing and uncovering new potential therapeutic targets for cervical cancer. It helps in discovering new treatment approaches, offering personalized precision treatment for patients, and aiding in the evaluation of disease progression and prognosis during treatment and follow-up.

### 3.3. LncRNA involved in HPV cervical cancer

Long noncoding RNAs (lncRNAs) are a subgroup of noncoding RNAs containing more than 200 nucleotides. Although they lack protein-coding ability, lncRNAs can regulate gene expression. LncRNAs are involved in key aspects of the occurrence, development, and progression of almost all different types of tumors.^[29,30]^ Many studies have shown that in both HPV-negative and HPV-positive cervical cancer, lncRNAs play a regulatory role, including in proliferation, invasion, apoptosis, and epithelial–mesenchymal transition.^[31–33]^

A study found that the tumor suppressor lncRNA NEF is specifically decreased in the tissues and serum of HPV-negative patients compared to healthy controls and HPV-positive cervical squamous cell carcinoma (SCC) patients. The study suggests that lncRNA NEF may inhibit the migration and invasion of HPV-negative SCC by suppressing the TGF-β pathway. As a key pathway, the TGF-β signaling pathway plays an important role in the occurrence and development of many human malignancies.^[34–36]^ Therefore, lncRNA NEF can help distinguish HPV-negative SCC from healthy individuals and HPV-positive SCC patients, and it can also serve as a potential therapeutic target for treating HPV-negative SCC. In postoperative follow-up of HPV-negative SCC patients, the low expression levels of lncRNA NEF in cervical tissues and serum are closely associated with poor survival rates.^[35]^ lncRNA NEF inhibits invasion and metastasis of HPV-negative SCC and can serve as a potential prognostic marker.

The long noncoding RNA Antidifferentiation ncRNA (lncRNA ANCR) is involved in cell differentiation in malignant tumors and has different functions in different types of malignancies. It plays a tumor-suppressing role in breast cancer, while promoting tumor invasion and metastasis in colorectal cancer.^[37,38]^ Hypoxia-inducible factor 1 alpha (HIF-1α) plays a crucial role in the occurrence and development of various malignancies in humans. Therefore, interventions targeting the HIF-1α pathway are a hot research topic in cancer treatment.^[39]^ Research has found that lncRNA ANCR inhibits tumor cell growth in HPV-negative SCC under hypoxic conditions by suppressing HIF-1α.^[32]^ In HPV-negative patients, cervical tissues and serum show significantly decreased or completely lost expression of lncRNA ANCR compared to normal females and HPV-positive SCC patients. This decrease is not influenced by age or alcohol consumption habits.^[32,36]^ This indicates that lncRNA ANCR has high stability, and the lack of expression or low expression of lncRNA ANCR can help screen for HPV-negative patients. In addition to changes in HPV-negative SCC, the expression of lncRNA ANCR varies in several types of malignancies.^[37,40]^ Therefore, in the diagnostic process, it is necessary to combine other biomolecules to exclude other malignancies.

Multiple studies have demonstrated that lncRNA HAND2-AS1 inhibits tumor cell growth, invasion, and metastasis in SCC.^[41–43]^ Researchers have found that lncRNA HAND2-AS1 not only regulates the VEGFA signaling pathway but also competitively binds to miR-21-5p to downregulate the expression of TIMP3, resisting cell apoptosis. It can also counteract the levels of C16orf74 by recruiting the transcription factor E2F4, thereby weakening cell colony formation, proliferation, invasion, and migration.^[43,44]^

There are many other tumor suppressor factors that play important roles. LncRNA DGCR5 and lincRNA 00173 inhibit cell proliferation through the Wnt signaling pathway and the miR-182-5p/FBXW7 axis, respectively. They play crucial roles in suppressing tumor invasion and metastasis in HPV-negative SCC.^[45,46]^ LncRNA PTCSC3 and lncRNA GAS5 exert tumor-suppressive effects by inducing cell apoptosis.^[47,48]^ LncRNA 00887 exerts its tumor-suppressive effects by inhibiting cell proliferation and invasion through the suppression of the miR-454-3p-mediated FRMD6-Hippo signaling pathway. On the other hand, low expression of lncRNA loc285194 is closely associated with a poor prognosis.^[49,50]^

LncRNA snaR inhibits HPV-negative SCC invasion and metastasis by promoting the degradation of TGF-β1. Moreover, distant recurrence of cervical cancer is associated with the downregulation or loss of lncRNA snaR.^[51]^ Conversely, lncRNA PVT1 exerts the opposite effect. By inhibiting TGF-β1 in tumor cells, it induces their proliferation, promotes tumor growth and metastasis. High expression of lncRNA PVT1 reflects a poorer prognosis.^[52]^ LINC00115, lncRNA MAGI2-AS3, lncRNA SOX2OT, lncRNA MALAT1, Linc00116, lncRNA XIST, and other lncRNAs, despite having different mechanisms of action, all play a promoting role in cancer development.^[36]^ The search for biomarkers and therapeutic targets is of great significance, as it helps provide more precise and personalized treatment for patients.

### 3.4. Other pathogenic mechanisms of HPV-negative cervical cancer

MiR-200a-3p, as a member of the miR-200 family, exerts either inhibitory or promotional effects on malignant tumors by regulating the calmodulin superfamily or Hippo signaling pathway.^[53,54]^ Yes-associated protein (YAP) is a key component of the Hippo signaling pathway and plays a promoting role in the development and progression of tumors.^[55]^ YAP is a downstream target of the Hippo signaling pathway and serves as a binding site for miR-200a-3p.^[56]^ In HPV-negative cervical cancer cells, upregulation of miR-200a-3p levels has been found to inhibit YAP expression, exerting a suppressive effect on tumor cell proliferation and invasion. However, the role of miR-200a-3p is opposite in HPV-positive cervical cancer.^[57]^ The miR-200a-3p-YAP signaling axis is of significant value in elucidating the mechanism of development and progression of HPV-negative cervical cancer. In clinical applications, it can be utilized for early diagnosis of cervical cancer, specific targeted drug development, disease progression monitoring, and prognosis evaluation to provide individualized precision treatment, ultimately improving patient outcomes.

In summary, the diagnosis of HPV-negative cervical cancer is often in the advanced stages, with a poorer prognosis compared to HPV-positive cervical cancer. Understanding its pathogenic mechanisms can help identify potential causes and triggers, which is significant for the development of preventive measures. Specific biomarkers discovered can be utilized to develop detection methods to increase the early diagnosis rate of HPV-negative cervical cancer. The molecular and signaling pathways involved in the pathogenesis serve as the foundation for specific drug development, aiding in the formulation of effective treatment strategies to improve the overall survival rate of these patients.

## 4. The pathological characteristics of HPV-negative cervical cancer tissue

The vast majority of cervical cancer is associated with HPV infection.^[2]^ Among them, HPV16 and HPV18 are the most common types of infection, followed by HPV31, 33, and 45.^[58]^ The proportion of HPV-negative cervical cancer varies according to different pathological types. Almost all SCC are associated with HPV infection, and HPV-negative SCC is extremely rare.^[14]^ Approximately 86% of cervical adenosquamous carcinomas are HPV-positive.^[59]^ While some subtypes of ADC are caused by non-HPV infections, accounting for approximately 15%.^[60]^ In cervical cancer, the HPV positivity rate varies according to different pathological types (Table 1). In ADC, the common, villoglandular, mucinous intestinal, and signet ring cell subtypes are common subtypes associated with HPV infection.^[14,16,59,61]^ The International Endocervical Adenocarcinoma Criteria and Classification (IECC criteria) classify endocervical adenocarcinomas into 2 major categories: HPV-related endocervical adenocarcinoma (HPVA) and non-HPV-related endocervical adenocarcinoma (NHPVA).^[62]^ Gastric-type adenocarcinoma is the most common type of NHPVA, while endometrioid adenocarcinoma is very rare (Table 2).

**Table 1:** HPV positivity rates in different pathological types.

Pathological types	HPV positivity rate
Squamous cell carcinoma	87% to 100%
Adenosquamous carcinoma	81% to 86%
Adenocarcinoma	
Usual type	80% to 100%
Villoglandular type	100%
Mucinous intestinal type	83% to 100%
Mucinous signet ring cell type	100%
Endometrioid type	0% to 27%
Mesonephric type	0%
Gastric type	0%
Clear cell type	0% to 28%
Serous type	0% to 30%

**Table 2:** IECC standard.

HPVA	NHPVA
Usual type	Gastric type
Villous adenocarcinoma	Mesonephric type
Mucinous type	Clear cell type
Mucinous, intestinal type	Serous type
Mucinous, signet ring cell type	Endometrioid type
Infiltrating mucinous carcinoma	Adenocarcinoma, nonspecific type

### 4.1. Cervical gastric-type adenocarcinoma

In the 1980s, the “gastric-type” cervical adenocarcinoma was first proposed in Japan. It is 1 of the HPV-independent pathological types and is the most common type of the second most common HPV-negative adenocarcinoma, accounting for approximately 10% of the total cases.^[63]^ This type of adenocarcinoma can be further subdivided into high-grade GCA to low-grade GCA. The mucinous type of well-differentiated adenocarcinoma also has a well-known name – malignant adenoma – which is a type of high-grade gastric-type adenocarcinoma.^[64]^ Under the microscope, a large amount of transparent or pale eosinophilic cytoplasm can be seen in tumor cells, along with moderate nuclear atypia and distinct cell borders, similar to adenocarcinoma cells of gastrointestinal origin.^[65]^ GCA tumor cells contain acidic mucin and express immunomarkers similar to gastric mucous cells, such as HIK1083, lysozyme, and pepsinogen II.^[66]^

The IECC classification categorizes GCA as HPV-independent cervical adenocarcinoma, considering the disease occurrence to be unrelated to HPV infection. p16 immunostaining is usually negative or focally positive, but still, 8% to 9% of cases may test positive for HPV infection. Some research has found that some cervical glandular adenocarcinoma cases exhibit a mixture of characteristics of gastric-type and usual-type adenocarcinoma.^[62,66–69]^ Selenica et al analyzed and detected mutations in 68 samples of GCA. They found that mutations in TP53 (41%), CDKN2A (18%), KRAS (18%), and STK11 (10%) were most common in tumor cells. They also discovered potential target mutations in ERBB3 (10%), ERBB2 (8%), and BRAF (4%). The study revealed that PIK3CA mutations were less frequent in GCA compared to typical adenocarcinoma.^[70]^ Lu et al conducted genetic testing on 15 GCA patients and found that TP53 (53%), STK11 (33%), CDKN2A (27%), ARID1A (20%), and PTEN (20%) were the most commonly mutated genes. They also observed frequent amplification of ERBB2 (13%). These mutations have an impact on the cell cycle and the PI3K/AKT signaling pathway.^[71]^ Similarly, Hodgson et al made similar findings in their study, where TP53 (46%), KRAS (36%), and PIK3CA (18%) were the most common mutated genes in GCA samples.^[72]^

### 4.2. Mesonephric adenocarcinoma

Middle tubular adenocarcinoma of the cervix accounts for <1% of all cervical adenocarcinomas and is a rare type of adenocarcinoma. It originates from remnants of the middle tubular duct or the middle tubular proliferation zone. Research has found that it is not associated with high-risk HPV infection.^[60,73]^ This type of tumor typically grows on the lateral to posterior wall of the cervix and can exhibit deep infiltration, nodular growth, or exophytic features. Histologically, middle tubular adenocarcinoma can be classified into tubular, ductal, solid, papillary, and reticular subtypes.^[74]^ Tubular structures consist of densely arranged small tubes composed of cuboidal cells. These tubes are tightly packed with low cytoplasmic content, and the lumen of the tubes contains eosinophilic colloid-like secretions. The nuclei are spherical, and differentiation of stromal hyperplasia is determined based on glandular aggregation, invasive growth, increased mitotic activity, intraluminal cell debris, and nuclear atypia.^[60]^

Middle tubular adenocarcinoma cells can express cytokeratins and epithelial membrane antigens such as E-cadherin, CD10, and PAX8. These adenocarcinomas are negative for estrogen and PRs as well as CEA, but they may express PAX8. Since their occurrence is not associated with HPV infection, strong positive p16 immunostaining reactions are typically not observed, although focal p16 expression may be present.^[75]^ Most patients with middle tubular adenocarcinoma exhibit typical KRAS mutations, with a very small subset carrying NRAS mutations. Neither PIK3CA nor PTEN mutations are detected in either primary or metastatic lesions, and microsatellite instability is not observed. However, chromosomal abnormalities such as increased copy number of 1q, loss of 1p, and gains on chromosomes 10 and 12 have been found in research studies.^[76]^ This type of tumor can recur and metastasize to distant sites, particularly the lungs, in the late stages, which is closely associated with the tumor’s invasiveness.^[77]^

### 4.3. Clear cell adenocarcinoma

In cervical glandular cancers, clear cell carcinoma accounts for approximately 3%. The morphology of clear cell adenocarcinoma (CCC) cells is similar to clear cell carcinomas of the endometrium or ovaries, and the structural composition includes solid, glandular cystic, and papillary components.^[60]^ The tumor cells have cytoplasm rich in glycogen, occasionally with hyaline globules.^[74]^ The cell nuclei are large and round, with highly recognizable features such as pleomorphism, prominent nucleoli, and increased chromatin content.^[62]^

The molecular mechanisms of CCC are currently not well understood. Current research suggests that its development may be associated with exposure to the synthetic estrogen hormone diethylstilbestrol (DES) used in intravaginal formulations.^[74]^ Boyd et al found that all CCC patients with a history of DES exposure exhibited microsatellite instability. In cases without DES exposure, approximately 50% showed microsatellite instability, but no mutations were found in the KRAS, HRAS, WT1, ER, or TP53 genes in these cases.^[78]^ In 3 cases of HPV-negative CCC (clear cell carcinoma), Jenkins et al identified 1 TP53 mutation and 1 PIK3CA mutation.^[28]^ Lee et al conducted genetic testing on a patient with cervical clear cell carcinoma and a history of intravaginal DES exposure. They found somatic mutations in the POLE and P286R genes in the patient’s somatic cells. Additionally, mutations in the PIK3CA, ARID1A, and PTEN genes were also detected.^[79]^ In immunohistochemical staining, no significant differences were found between cervical, endometrial, and ovarian clear cell carcinomas. Cervical clear cell carcinomas often show negative ER and PR expression, positive expression of PAX8, HNF1-beta, and Napsin-A, and variable expression levels ranging from negative to diffuse and strong positive for p16 and p53.^[75]^

Although there is no direct evidence linking CCC to endometriosis, studies have found that adjacent tubal-endometrioid implants may represent a potential precursor to CCC.^[80]^ This subtype of adenocarcinoma (clear cell carcinoma) is more aggressive in terms of recurrence and metastasis compared to SCC. Local recurrence may be more common than distant organ metastasis.^[74,81,82]^ The lungs, liver, and bones are the most common sites for extrapelvic metastasis of clear cell carcinoma.^[83]^

### 4.4. Serous carcinoma

Primary cervical serous carcinoma is extremely rare, and its classification and origins are still controversial. On 1 hand, “serous carcinoma” may represent a morphological variant of HPV-positive common type endocervical adenocarcinoma. On the other hand, “serous carcinoma” may potentially be a metastasis from ovarian, fallopian tube, or uterine serous carcinoma, misdiagnosed as primary cervical cancer.^[60,84]^ In terms of histological structure, serous carcinomas often present with a papillary architecture, with papillary projections, and are composed of stratified and pseudostratified cells with diffusely abnormal, hyperchromatic nuclei. Sometimes, psammoma bodies may also be formed.^[60,75]^ The research on patients with cervical serous carcinoma found that the age of onset has a bimodal distribution. Postmenopausal patients are HPV-negative and WT1-positive, with a poor prognosis, while premenopausal patients are HPV-positive and WT1-negative, with an overall favorable prognosis.^[85,86]^ Jenkins et al conducted genetic and immunohistochemical analyses on 45 cases of cervical adenocarcinoma. They found that common mutated genes in HPV-negative cervical serous adenocarcinoma include TP53, KRAS, PIK3CA, and PTEN.^[28]^ After clearly identifying the true origin of the tumor, the treatment target can be determined. Studies have found that in 30% of cervical serous adenocarcinomas, the HER2/neu receptor is positive. Adding trastuzumab to the chemotherapy regimen can extend the survival of these patients by 3 to 8 months.^[87]^

### 4.5. Endometrioid adenocarcinoma

Primary endocervical endometrioid adenocarcinoma is a type of adenocarcinoma with endometrioid morphological features. The tumor cells lack mucin and have low cytoplasmic content, appearing eosinophilic.^[74]^ The nuclei are arranged in a pseudostratified pattern, and the cellular atypia is usually not more than moderate. This type of tumor may or may not be accompanied by squamous differentiation and endometrial ectopia. The occurrence of this tumor is not significantly related to HPV infection.^[60]^ HPV infection is not significantly associated with the occurrence of this type of tumor.^[88]^ In all cervical glandular cancers, endocervical adenocarcinoma accounts for <5%.^[89]^ After improving detection methods and diagnostic criteria, the percentage is now only 1%.^[74]^ There are reports that endocervical-like adenocarcinoma of the cervix originates from endometriosis of the cervix.^[90,91]^ Therefore, before diagnosing endometrioid adenocarcinoma, it is necessary to differentiate it from typical cervical glandular adenocarcinoma and endometrial cancer involving the cervix.

Due to the rarity of endocervical-like adenocarcinoma of the cervix, research on its molecular genetic characteristics is still in the early stages. Recently, Jenkins et al conducted genetic testing on 8 HPV-negative patients with endocervical-like adenocarcinoma and found mutations in various somatic genes, including PIK3CA (50%), PTEN (50%), CTNNB1 (37.5%), FBXW7 (25%), KRAS (12.5%), AKT1 (12.5%), and MSI-H (12.5%). MSI-H can serve as a potential target for targeted therapy in clinical practice.^[28]^

## 5. Clinical characteristics of HPV-negative cervical cancer

HPV-negative cervical cancer, due to its relatively indolent onset, is prone to progress to advanced FIGO stages and lymphovascular invasion before diagnosis, leading to a poor prognosis.^[9,92]^ A global study showed that 83.7% of common types of cervical cancer are diagnosed at stage I, whereas HPV-negative adenocarcinomas are diagnosed at stage II or higher in 62.5% of cases.^[93]^ The average age at diagnosis for patients with HPV-negative cervical cancer is higher than that of HPV-positive patients. Research indicates that the average age at diagnosis for HPV-positive cervical cancer patients is 49 ± 13.3 years, while for HPV-negative patients, it is 62.4 ± 14.9 years.^[94]^ Different types of HPV-negative cervical cancer have unique characteristics. Gastric-type adenocarcinoma is more common in the upper part of the cervix, and excessive vaginal bleeding and watery discharge are common symptoms. Due to its invasive growth, the most prominent physical sign on examination is enlargement of the cervix without palpable masses.^[95]^ Clear cell carcinoma presents with abnormal vaginal bleeding, is unresponsive to hormone therapy, and on examination, cervical erosion is observed.^[96]^ Middle tubular adenocarcinoma is more commonly found in the lateral to posterior aspects of the cervix, with very few lesions involving the entire cervix. The tumor often has unclear borders and can exhibit infiltrative, nodular, or exophytic growth patterns.^[73]^

HPV-negative cervical glandular adenocarcinoma exhibits a higher propensity for aggressive invasion with bleeding compared to other common cervical cancer types. As a result, it is more prone to recurrence and distant metastasis. Common metastatic sites include the lungs, liver, and bones.^[83,97–99]^ Multiple studies on cervical cancer have found that HPV-negative cervical cancer is associated with a poor prognosis. A 10-year follow-up study of 204 cervical cancer patients showed that the 5-year survival rates for HPV-negative and HPV-positive patients were 82% and 58%, respectively (*P* = .003).^[100]^ The disease-free survival periods were 132.2 months (95% CI: 32.0–87.6 months) for HPV-negative patients and 59.8 months (95% CI: 118.6–145.8 months) for HPV-positive patients, with a statistically significant difference (*P* < .01).^[9]^ Existing studies indicate that among different types of HPV-negative cervical glandular adenocarcinoma, survival rates are lower for endometrioid adenocarcinoma and gastric-type adenocarcinoma.^[77,97]^

## 6. Conclusion

Although HPV-negative cervical cancer accounts for only 3% to 8% of cervical cancer cases, its characteristics such as hidden onset, strong invasiveness, and poor prognosis should be given serious attention. Currently, our research on cervical cancer is mainly focused on HPV-positive cases, and further research on HPV-negative cases is needed. Due to reasons such as tumor classification, detection techniques, and sampling, false-negative results often occur, so it is necessary to continuously improve the sensitivity of HPV detection, standardize specimen collection procedures, and subdivide pathological tissue types. The pathogenesis of HPV-negative cervical cancer is still in the research stage, and it is believed to be related to the immune microenvironment, tumor gene mutations, and some lncRNA. With an increasing number of reports on these patients, clarifying the pathogenic mechanism is imminent, and designing research models targeting HPV-negative cervical cancer will be an important part of future research. The discovery of highly sensitive biomarkers will help increase the number of early diagnoses of HPV-negative cervical cancer, and identifying specific molecules and disease-causing gene pathways is the cornerstone of developing specific targeted drugs and personalized treatments.

## Author contributions

**Conceptualization:** Ning Shao.

**Data curation:** Ning Shao.

**Formal analysis:** Ning Shao.

**Funding acquisition:** Ning Shao.

**Investigation:** Ning Shao.

**Methodology:** Ning Shao.

**Project administration:** Ning Shao.

**Resources:** Ning Shao.

**Software:** Ning Shao.

**Supervision:** Ning Shao.

**Validation:** Ning Shao.

**Visualization:** Ning Shao.

**Writing – original draft:** Ning Shao.

**Writing – review & editing:** Ning Shao.
